# Simultaneous ^18^F-FDG PET/MR metabolic and structural changes in visual snow syndrome and diagnostic use

**DOI:** 10.1186/s13550-022-00949-0

**Published:** 2022-12-30

**Authors:** Koen Van Laere, Jenny Ceccarini, Juanito Gebruers, Karolien Goffin, Elizabet Boon

**Affiliations:** 1 Nuclear Medicine, University Hospitals Leuven, UZ Leuven, Campus Gasthuisberg, Nucleaire Geneeskunde, E901, Herestraat 49, 3000 Leuven, Belgium; 2 Nuclear Medicine and Molecular Imaging, Department of Imaging and Pathology, KU Leuven, Leuven, Belgium; 3 Leuven Brain Institute (LBI), KU Leuven, Leuven, Belgium; 4 Division of Neurology and Psychiatry, University Hospitals Leuven and UPC Kortenberg, Leuven, Belgium

**Keywords:** Visual snow syndrome, ^18^F-FDG PET, Voxel-based morphometry, PET/MR, Classification accuracy

## Abstract

**Purpose:**

Visual snow syndrome (VSS) is a recently recognized chronic neurologic condition characterized by the constant perceiving of tiny flickering dots throughout the entire visual field. Metabolic overactivity and grey matter volume increase in the lingual gyrus has been reported. We investigated this by ^18^F-FDG PET/MR in comparison to healthy controls. Aside from voxel-based characterization, the classification accuracy of volume-of-interest (VOI)-based multimodal assessment was evaluated, also in comparison with visual analysis.

**Methods:**

Simultaneous ^18^F-FDG PET and MR imaging was performed in 7 patients with VSS (24.6 ± 5.7 years; 5 M/2F) and 15 age-matched healthy controls (CON) (28.0 ± 5.3 years; 8 M/7F). SPM12 and voxel-based morphometric analysis was performed. A VOI-based discriminant analysis was performed with relative ^18^F-FDG uptake, MR grey matter (GM) volumes and their combination. A visual analysis was done by two blinded experienced readers.

**Results:**

Relative increased hypermetabolism was found in VSS patients in the lingual gyrus and cuneus (*p*_FWE_ < 0.05, peak change + 24%), and hypometabolism in the mesiotemporal cortex (*p*_height,uncorr_ < 0.001, peak change − 14%). VSS patients also had increased GM volume in the limbic system and frontotemporal cortex bilaterally (*p*_FWE_ < 0.05), and in the left secondary and associative visual cortex and in the left lingual gyrus (*p*_height,uncorr_ < 0.001). Discriminant analysis resulted in 100% correct classification accuracy for ^18^F-FDG with lingual gyrus, cuneus and lateral occipital lobe (BA 17 and BA 18) as main discriminators. Unimodal MR- and combined ^18^F-FDG + MR classification resulted in an accuracy of 91% and 95%, respectively. Visual analysis of ^18^F-FDG was highly observer dependent.

**Conclusion:**

Patients with VSS have highly significant structural and metabolic abnormalities in the visual and limbic system. VOI-based discriminant analysis of ^18^F-FDG PET allows reliable individual classification versus controls, whereas visual analysis of experienced observers was highly variable. Further investigation in larger series, also in comparison to VSS mimicking disorders such as migraine, is warranted.

*Trail registration*: Retrospectively registered at clinicaltrials.gov under NCT05569733 on Oct 5, 2022.

**Supplementary Information:**

The online version contains supplementary material available at 10.1186/s13550-022-00949-0.

## Introduction

While negative visual disturbances such as blindness and visual field defects are promptly recognized, positive visual disturbances such as distortion of a real visual sensory stimulus (illusion) or the perception of a visual image without the existence of a visual stimulus (visual hallucination), are more difficult to diagnose. Visual hallucinations can consist of formed images (people, objects), but also unformed images including geometric designs, scintillating scotomas and visual snow (VS) [1, 2]. In VS, patients present with a continuous, pan field “snow” like static of an analog television: tiny, dynamic, usually black and white dots filling the entire visual field. The condition was first reported in 1995 [3], but has only recently been defined as a consensus “visual snow syndrome” (VSS) [4]. Aside from continuous VS for longer than 3 months, a diagnosis of VSS requires at least two additional visual symptoms of palinopsia (inability to suppress the just-seen), enhanced entoptic phenomena (inability to suppress the visualization of the optic apparatus), nyctalopia or photophobia. VSS is an independent entity from migraine and aura, yet approximately 60% of patients with VSS also experience concomitant migraine [4–6]. The disorder is rare, mostly occurring in young adulthood with mean age of onset in the third decade [1], yet a recent community-based study reported prevalence up to 2% [7]. VSS can be highly disabling due to the continuous presence of visual symptoms as well as the lack of treatment options and objective measures to differentiate the disorder from malingering or psychogenic visual disturbance. Treatment options are limited and have only fair success, including use of colorimetric lenses and pharmacologic interventions with oral diuretics (acetazolamide), anticonvulsants (lamotrigine) and antidepressants; in severe cases greater occipital nerve block can be done with complete response in 50% [1, 8, 9].

In the past few years, VSS has been characterized in more detail by clinical, neuroimaging and neurophysiologic investigations [10–16]. VSS seems to involve aberrant processing of visual information in the supplementary visual cortex, and there is increasing evidence that VSS involves multiple mechanisms with cortical dysfunction also outside the visual system. Cortical hyperexcitability, due to increased neural contrast gain rather than abnormal neural noise [17], changes in specific visual streams, altered thalamocortical pathways and dysfunction of higher-level salience network controls have been suggested [18]. Interestingly, tinnitus, considered the auditory analogue to visual snow, is also highly prevalent in VSS, which suggests a common pathway [19].

Patients usually present with normal ophthalmological, neurological and regular structural MR imaging examinations [1, 4, 16, 20]. However, using brain ^18^F-FDG PET, volumetric MRI and advanced MRI sequences, highly consistent changes have been observed in group comparisons, suggesting that in vivo neuroimaging biomarkers may hold potential as objective measures of VSS. In a landmark study, first published in 17 patients [21] with later extension to 20 patients [13], Schankin et al. showed that VSS patients have cerebral hypermetabolism within the (right) lingual gyrus, as well as hypometabolism in the right superior temporal gyrus and the left inferior parietal lobule. Structural MRI-based voxel-based morphometry showed cortical grey matter volume (GMV) increase in the extrastriate visual cortex at the junction of the right lingual and fusiform gyrus [13, 22]. The latter finding was independently confirmed by Aldusary et al. [23], showing extended GMV increase in the early and higher visual cortex as well as the temporal cortex, with an association between GMV increase in the lingual gyrus and disease duration. Puledda et al. also reported metabolic and functional alterations using MR spectroscopy with elevated lactate concentration of the (right) lingual gyrus and disturbed processing in the insular cortex (salience network) on task-based fMRI [14]. In an arterial spin labeling MR study in 24 subjects, VSS patients had higher regional blood flow (rCBF) than controls over an extensive brain network, including the cuneus, precuneus and fusiform gyrus bilaterally with a decrease in right insula rCBF [24]. Finally, diffusion changes in white matter (WM), tracts associated with visual processing and conceptualization such as the inferior fronto-occipital fascicle, temporal and occipital WM have been observed in VSS, although but it is unclear how specific this is versus migraine [25].

However, it remains to be demonstrated if (some of) these pathophysiological alterations can be translated towards clinical utility. In order to evaluate the value of PET and volumetric MR imaging in a clinical diagnostic context, we prospectively evaluated a patient group fulfilling the diagnostic criteria of VSS [4] using simultaneous PET/MR, in comparison to an existing database of carefully screened, age-matched healthy controls. Aside from a voxelwise group comparison, we also evaluated the classification accuracy of a VOI-based discriminant analysis of ^18^F-FDG and GMV, as well as visual ^18^F-FDG PET analysis.

## Materials and methods

### Participants

We included 7 patients (24.6 ± 5.7 years, 5 M/2F) diagnosed with VSS. All patients were referred for imaging by a neurologist (E.B.) specialized in migraine and VSS. Other underlying neurological conditions were excluded and ophthalmological examinations were performed to exclude other underlying ophthalmological conditions. Inclusion criteria were visual snow with dynamic, continuous, black and white tiny dots in the entire visual field lasting longer than 3 months, with at least two additional visual symptoms [4]. All patients completed a questionary that focused on the presence of continuous or episodic visual snow, additional visual symptoms (palinopsia, entopic eye phenomena, photophobia and nyctalopia), the beginning of their visual symptoms, association of migraine, migraine aura and tinnitus, their general current and past medical history, and illicit drug use [26]. Patients were asked to participate at referral to undergo a subsequent PET/MR investigation after their routine FDG PET/CT.

An age- and gender-matched subset of 15 healthy controls (CON) (mean age 28.0 ± 5.3 years; 8 M/7F) was randomly selected from a large normal ^18^F-FDG PET/MR database obtained on the same imaging system [27]. The main relevant exclusion criteria were history of major internal pathology, neurological and/or psychiatric disorders (including psychosis, depression, and anxiety), history of frequent migraine attacks, substance abuse or current use of any central acting medication, first-degree relatives with dementia. All CON had a normal neurological examination, Mini-Mental State Examination (MMSE) ≥ 28, Beck’s Depression Inventory (BDI) score ≤ 9, and a normal structural T1 and T2 MRI.

The study was approved by the KU Leuven Ethics Committee under study numbers S58764 (patients) and S58571 (control data set). The study was conducted in accordance with the ethical standards as laid down in the Declaration of Helsinki and its later amendments. All subjects signed written informed consent before enrollment in the study.

### Image acquisition

All subjects fasted at least for 4 h prior to ^18^F-FDG injection. ^18^F-FDG was injected intravenously (149 ± 10 MBq) in standard ambient conditions, supine in a dark, noise free room with eyes and ears open. ^18^F-FDG PET images were acquired for 20 min on a simultaneous GE Signa 3T PET/MR scanner with integrated Time-of-Flight (TOF) (GE Healthcare, Chicago, IL, USA), starting 64.7 ± 10.8 min postinjection, as routine ^18^F-FDG PET/CT was first acquired in routine setting after 30 min. For CON, dynamic PET data were acquired for 60 min starting from ^18^F-FDG injection (146 ± 9 MBq). From the list-mode data, the last 20-min were reconstructed and used as comparator for this study.

Simultaneous with the ^18^F-FDG PET/MR acquisition, zero-echo-time (ZTE) MR (3D radial acquisition; Flip Angle: 0.8°; Bandwidth: 62.5 kHz) images for attenuation correction [20] and a 3D volumetric T1-weighted BRAVO MR sequence [echo time (TE) = 3.2 ms; repetition time (TR) = 8.5 ms; voxel size = 1 × 1 × 1 mm] were acquired, using a vendor supplied high-resolution 8-channel phased array head coil (GE Healthcare, Milwaukee, USA).

The 20-min ^18^F-FDG PET list mode data were rebinned in 4 frames of 5 min, corrected for deadtime, randoms, scatter and time-offset. A previously validated MR-based attenuation correction using the ZTE MR images was applied [28]. PET images were reconstructed using OSEM (ordered subset expectation maximization; 28 subsets, 4 iterations), including TOF information, resolution modelling and smoothed with in-plane Gaussian kernel with a FWHM (full width at half maximum) of 4.5 mm.

### ^18^F-FDG PET image processing

^18^F-FDG PET frames were first corrected for motion by a rigid frame-by-frame coregistration and a single static ^18^F-FDG image was obtained as the average of all motion-corrected frames using PMOD software (v4.1; PMOD Inc. Zürich, Switzerland). ^18^F-FDG data were coregistered to the individual volumetric T1-weighted MR images and were then analyzed quantitatively by voxel- and volumes-of-interest (VOI)-based analyses. Before performing the voxel-based group comparison analysis, all the coregistered ^18^F-FDG PET were spatially normalized using the nonlinear deformation fields generated by the CAT12 toolbox using a DARTEL template (voxel size: 1.5 × 1.5 × 1.5 mm), and subsequently smoothed using a Gaussian kernel with FWHM of 8 mm. PET images were analyzed using proportional scaling to the average GM activity.

### Voxelwise and semiquantitative VOI-based ^18^F-FDG PET analysis

To assess the differences in glucose metabolism between the VSS and CON group, a whole-brain Statistical Parametric Mapping (SPM12; Welcome Trust Centre for Neuroimaging, University College, London, UK) implemented in Matlab (R2020b, The MathWorks Inc., Natick, MA, USA) group comparison was carried out [two-sample independent t-test; significance level set to *p*_FWE_ (familywise error corrected) < 0.05 at cluster level, *p*_height-FWE_ < 0.05 unless stated otherwise, and extent threshold *K*_EXT_ > 200 voxels approximately 0.68 ml)]. As this analysis was considered exploratory, we also applied a lower threshold of significance, *P*_height_ < 0.001 uncorrected at cluster level. An explicit binary mask was created by first averaging the individual normalized GM and CSF probability maps, and subsequently by subtracting the binarized averaged CSF mask from the binarized averaged GM mask. Also, based on the possible effect of the comorbid conditions migraine and tinnitus, additional statistical designs were performed with binarized presence of migraine or tinnitus symptoms as covariates.

VOI-based analysis was performed using the PMOD PNEURO tool (v4.1; PMOD Inc. Zürich, Switzerland) and the N30R83 Hammers probability atlas [22] resulting in 83 automatically delineated brain VOIs. Individual VOI ^18^F-FDG activities were normalized to the individual average grey matter (GM) activity to obtain the relative VOI metabolic activity. Aside from lingual gyrus, cuneus and lateral occipital cortex which were the main focus based on previous literature, the other regions were grouped into 9 larger bilateral composite regions: frontal cortex, cingulate cortex, temporal cortex, mesotemporal cortex, parietal cortex, (total) occipital cortex (lingual gyrus, cuneus and lateral occipital cortex), striatum, thalamus, insula and cerebellum. VOI-based group comparisons were assessed using a two-tailed unpaired *t* test and Bonferroni post-hoc tests (*p* < 0.05/12 = 0.004).

### Voxel-based morphometry and MR volumetry

MR volumetric differences between VSS and CON were assessed both at the voxel-level (voxel-based morphometry, VBM) and at the VOI level. Individual specific tissue probability maps for GM, WM and cerebrospinal fluid (CSF) were obtained by segmenting the spatially normalized 3D T1-weighted MR scans using the CAT12 toolbox (standard setting for parameters) of SPM12. The modulated warped GM probability maps were first smoothed with a kernel with 8 mm FWHM and then used as input for the VBM analysis.

For VBM, the preprocessed images were entered into a statistical unpaired t-test design, with total intracranial volume (TIV) as a nuisance covariate to correct for different brain sizes. To exclude extracerebral clusters, the same explicit binary GM mask as used for the voxel-based ^18^F-FDG PET analysis was applied. Data were explored at *p*_cluster_ < 0.05, and two thresholds were used at the voxel-level: a stringent *p*_height-FWE_ < 0.05 and a lower exploratory threshold *p*_height_ < 0.001 uncorrected. The cluster extent (*k*_ext_) level was set at 200 voxels.

For the VOI-based analysis, GMV values were also extracted from the Hammers atlas after segmentation of the T1-weighed MR images within native MRI space in the PMOD PNEURO tool, and grouped into the same 12 larger composite VOIs as used for ^18^F-FDG. The GM VOI values were subsequently normalized to the TIV. VOI-based group comparisons were assessed using a two-tailed unpaired t-test and Bonferroni post-hoc tests (*p* < 0.05/12 = 0.004).

### Descriptive statistics and discriminant analysis.

Descriptive statistics were performed with GraphPad Prism 9.1 (GraphPad Software, La Jolla, CA, USA). Significance was accepted at the 95% probability level.

Discriminant analysis was performed using SPSS Statistics v26.0 for Windows (IBM Armonk, NY, USA) with general discriminant modeling. The independent variables (*predictor variables*) used to predict the *grouping variable* (CON vs. VSS) were regional relative VOI uptake data for ^18^F-FDG and MR GMV values. VOI data were entered independently into the discriminant function at once. For all analyses, a leave-one-out post-hoc classification was performed, only these data are reported. Receiver operating characteristic (ROC) analysis was performed for the significant discriminant ^18^F-FDG and MR GM VOIs to assess the diagnostic accuracy in discriminating between the VSS and CON groups.

### Visual analysis of ^18^F-FDG PET

Prior to visual analysis, all ^18^F-FDG scans were fully anonymized, randomized and spatially normalized and processed using MIM-Neuro® as done in our clinical setting with transverse, sagittal and coronal slices and a 3D surface rendering of the PET data (v7.0; MIM software Inc., Cleveland, OH, USA) made available to the observers. Two experienced nuclear medicine physicians (K.V.L. and K.G., with 25 and 10 years of experience in brain imaging, respectively) visually analyzed and rated all images in a blinded fashion unaware of clinical information at time of the scan. The following instructions were given: firstly, to score activity in relevant regional left and right predefined areas (frontal, temporal, medial temporal, parietal, occipital, primary and secondary visual cortex, lingual gyrus, striatum, thalamus, and cerebellum), by using a 5-point scale (− 2 = *strongly decreased*, − 1 = *slightly decreased*, 0 = *normal*, 1 = *slightly increased*, 2 = *strongly increased*). Secondly, the observers were asked to binary classify the subject as “VSS patient” or “CON” with knowledge of previous literature data of Schankin [13], the SPM result in our group analysis and the number of subjects in each group. Finally, observers also gave a confidence rating for their binary classification, scaled as: 1 = *very uncertain*, 2 = *rather uncertain*, 3 = *reasonably certain*, 4 = *certain*, and 5 = *very certain*. The diagnostic accuracy (sensitivity, specificity, and accuracy) was calculated for each observer by direct comparison to the ground truth. Visual assessment of the MRI data was not attempted since the GM changes were deemed too subtle for visual detection conform earlier literature [1, 4, 16, 20].

## Results

### Patient demographical and clinical parameters

An overview of demographical and clinical parameters of the patients with VSS is provided in Table 1. Seven patients (5 M/2F, age 24.6 ± 5.7 years) were included in the study. Patients had an average disease duration of 36 ± 25 months, ranging from 8 to 72 months. All VSS patients reported black and/or white VS, and at least two additional visual symptoms (5 of 7 had at least three additional visual symptoms). Enhanced entopic phenomena (floaters, blue field phenomenon, photopsia or self-light) were reported by all patients, palinopsia by 6 patients, photophobia by 5 patients and nyctalopia by 4 patients (Table 1).

**Table 1:** Patients characteristics and clinical parameters

	Age (y)	Age at onset (y)	Sex	VS	Visual symptoms				Non-visual symptoms			Psychiatric symptoms
					Palinopsia	Enhanced entopic phenomena	Photophobia	Nyctalopia	Comorbid tinnitus	Comorbid migraine	Comorbid migraine aura	
Patient 1	18	16	F	1	1	1	0	0	0	1	0	D
Patient 2	34	28	M	1^a^	0	1	1	1	1	1	1	0
Patient 3	24	23	M	1	1	1	1	1	1	1	1	A, D
Patient 4	26	23	M	1	1	1	1	0	1^c^	0	0	A, D, Dp, P
Patient 5	29	28	F	1^b^	1	1	0	0	1	0	0	CN
Patient 6	22	17	M	1	1	1	1	1	0	1	1	A, D, Dp
Patient 7	19	16	M	1	1	1	1	1	1	1	1	A, D, Dp

*VS* visual snow symptom, Psychiatric symptoms: *A* anxiety, *D* depression, *Dp* depersonalization, *P* psychosis, *CN* compulsive neurosis, *M* male, *F* female

^a^Only in dark and when closing the eyes

^b^Only in the morning

^c^In the context of early noise pollution

Moreover, migraine and tinnitus were the most frequently reported comorbid non-visual symptoms in 5 of 7 subjects, with 4 of these 5 having migraine with migraine aura. Anxiety, depression or depersonalization was reported by 5 patients. Patients reported a low weekly consumption of alcohol (< 5 units/week; 4 patients) or full abstention of alcohol (3 patients). Prior lifetime cannabis or recreational drug use was reported by 5 subjects, with at least 8 months (range 8–156 months) passed from their last use. One patient (#7, Table 1) indicated he was still a sporadic cannabis user, two patients were completely drug-naïve. No formal drug tests (saliva, hair, urine) were carried out before imaging.

### Voxel-wise and VOI ^18^F-FDG PET analysis

**Fig. 1:**
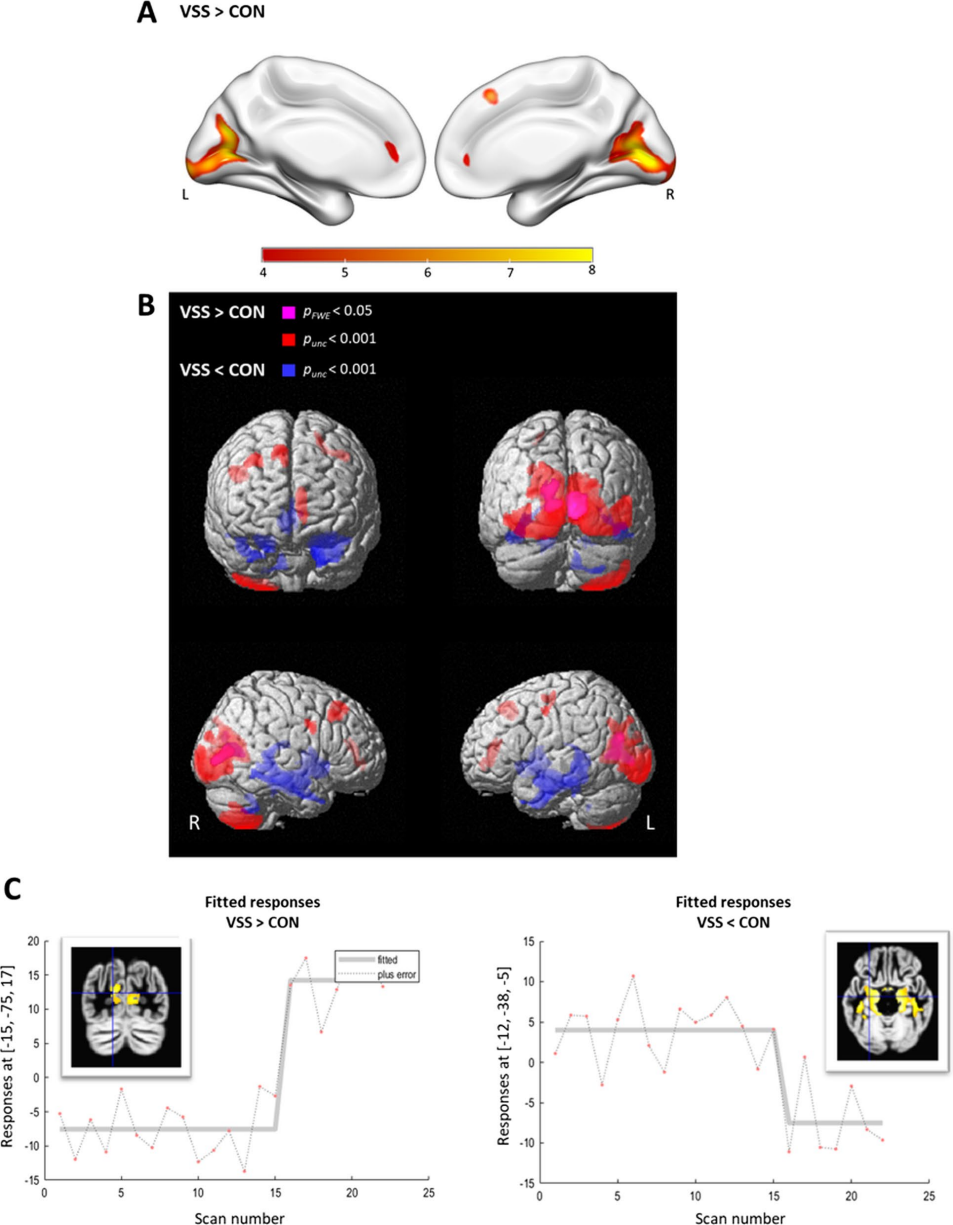
T-statistical map of significant clusters in the voxel-based two sample *t* tests of ^18^F-FDG PET of visual snow syndrome patients (VSS) versus healthy controls (CON). **A** Red–yellow scale shows increased glucose metabolism in VSS compared to CON. The color bar represents *t* values for SPM group comparison at *p*_FWE_ < 0.05 corrected for multiple comparisons on voxel level (*k*_ext_ = 200 voxels). **B** Surface rendering with hypermetabolic (VSS > CON; purple: *p*_FWE_ < 0.05; red: *p*_height,uncorrected_ < 0.001) and hypometabolic (VSS < CON; blue: *p*_height,uncorrected_ < 0.001) regions in VSS compared to CON. L = left, R = right. **C** Plot of the effect in the visual cortex (left; peak voxel Talairach coordinate [− 15, − 75, 17 = L calcarine], see Table 2) and medial temporal cortex (right; peak voxel Talairach coordinate [− 12, − 38, − 5 = L parahpc gyrus], see Table 2) shows no overlap; CON scan number 1–15 and VSS 16–22

In VSS patients, clusters of significant relative hypermetabolism were observed in the primary and secondary visual cortex bilaterally (BA17 and BA18), including the calcarine cortex, lingual gyrus and cuneus, with spreading towards the associative visual cortex (BA19) (*p*_FWE-corr_ < 0.0001 at cluster level, range T-values: 8.4–11.7; + 24% peak voxel intensity difference; Fig. 1, Table 2). At a lower *p*-level (*p*_uncorr_ < 0.001), one additional cluster of relative hypermetabolism was observed in VSS covering the cranial right cerebellar lobules 7b and 8 (Fig. 1B, Table 2). These results remained unchanged when presence of migraine and tinnitus were added as additional covariate variable.

**Table 2:** Results of the voxel-based group comparison (SPM12) showing the significant glucose metabolism differences in visual snow syndrome (VSS) patients compared to healthy controls (CON)

Cluster level		Voxel level		Peak voxel Talairach coordinate			Cluster peak intensity	Cluster location
*P*_FWE-corr_	*K*_ext_	*P*_height_ (FWE)	*T* score	*X*	*Y*	*Z*	*Δ*% (%)	Anatomical region
*(A.1) VSS* > *CON (p*_*height-FWE*_ < *0.05 at voxel-level)*								
< 0.0001	656	< 0.0001	11.7	− 15	− 75	17	+ 24	L calcarine (BA18)
		0.001	8.7	− 12	− 68	8		L lingual gyr (BA17)
< 0.0001	970	0.001	8.6	9	− 69	12	+ 24	R calcarine (BA17)
		0.002	8.4	8	− 83	3		R calcarine (BA17)
*(A.2) VSS* > *CON (p*_*height-uncorr*_ < *0.001 at voxel-level)*								
< 0.0001	11,494	< 0.0001	11.7	− 15	− 75	17	+ 24	L Cuneus (BA18)
		< 0.0001	8.7	− 12	− 68	8		L calcarine (BA17)
		< 0.0001	8.6	9	− 69	12		R calcarine (BA17)
		< 0.0001	8.0	− 12	− 101	− 3		L occip pole (BA17)
		< 0.0001	7.0	− 2	− 87	0		L lingual gyr (BA17)
		< 0.0001	6.8	50	− 75	− 6		R inf occip gyr (BA19)
0.004	1277	< 0.0001	6.6	45	− 63	− 53	+ 37	R CBL_VIIb
		< 0.0001	6.4	38	− 60	− 60		R CBL_VIII
*(B) VSS* < *CON (p*_*height-uncorr*_ < *0.001 at voxel-level)*								
< 0.0001	12,006	< 0.0001	6.3	− 12	− 38	− 5	− 14	L parahpc gyr
		< 0.0001	6.2	20	− 18	− 23		R parahpc gyr
		< 0.0001	6.0	− 15	− 11	− 17		L HpC
		< 0.0001	5.9	33	− 38	2		R HpC

At voxel level *p*_height-cluster FWE-corr_ < 0.05 (A.1) and *p*_height-cluster uncorr_ < 0.001 (A.2 and B) with cluster extent 200 voxels

*BA* Brodmann area, *CBL* cerebellum, *HpC* hippocampus, *parahpc* parahippocampus, *L* left, *R* right

Inversely, a cluster of significant hypometabolism was observed in the mesotemporal cortex bilaterally, including the hippocampus and parahippocampal gyrus (*p*_uncorr_ < 0.0001, range *T* values: 5.9–6.3; − 14% intensity difference at the peak; Fig. 1B, C, Table 2). The same hypometabolic cluster was obtained after adjusting for the presence of migraine only (Additional file 1: Fig. S1). By adding the two covariates migraine and tinnitus into the analysis model, the cluster of decreased metabolism was smaller and only present at reduced statistical threshold of *p*_uncorr_ < 0.005 (Additional file 1: Fig. S1).

**Fig. 2:**
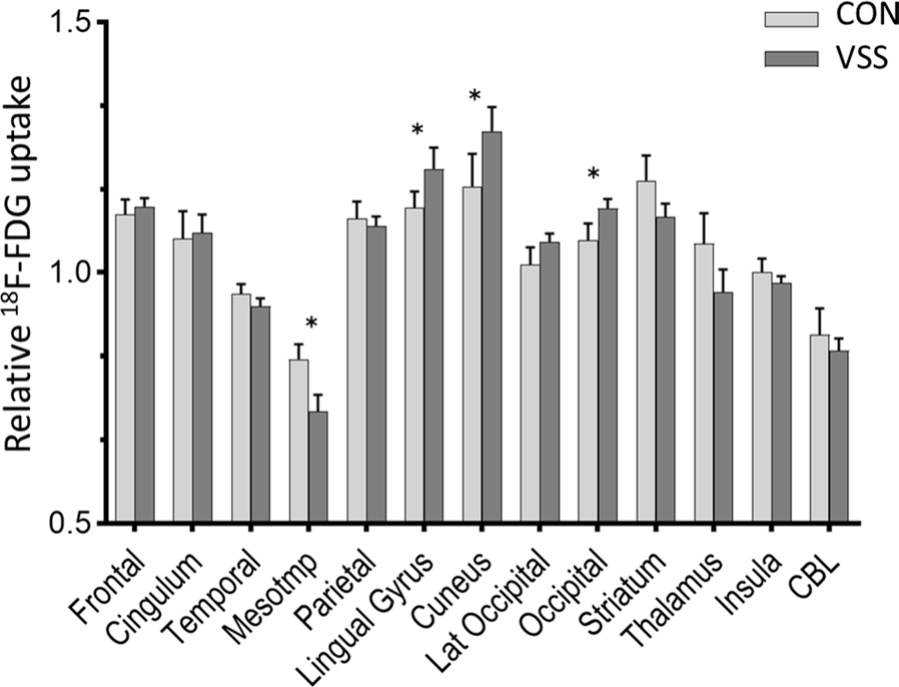
Relative ^18^F-FDG uptake in 13 composite VOIs in visual snow syndrome subjects (VSS) versus healthy controls (CON). *Indicates *p* < 0.004 (after Bonferroni correction); CBL cerebellum; occipital = composite VOI of lingual, cuneus and lateral occipital VOIs

These findings were confirmed by the VOI analysis, with a higher relative glucose metabolism in VSS patients in the cuneus (+ 9.5%), lingual gyrus (+ 6.9%) and occipital cortex (+ 5.9%) VOIs (average increase over the VOI), together with a lower glucose metabolism in the medial temporal cortex VOI (− 12.5%) (all *p* < 0.004, Bonferroni corrected) (Fig. 2).

### Voxel-based morphometry

**Fig. 3:**
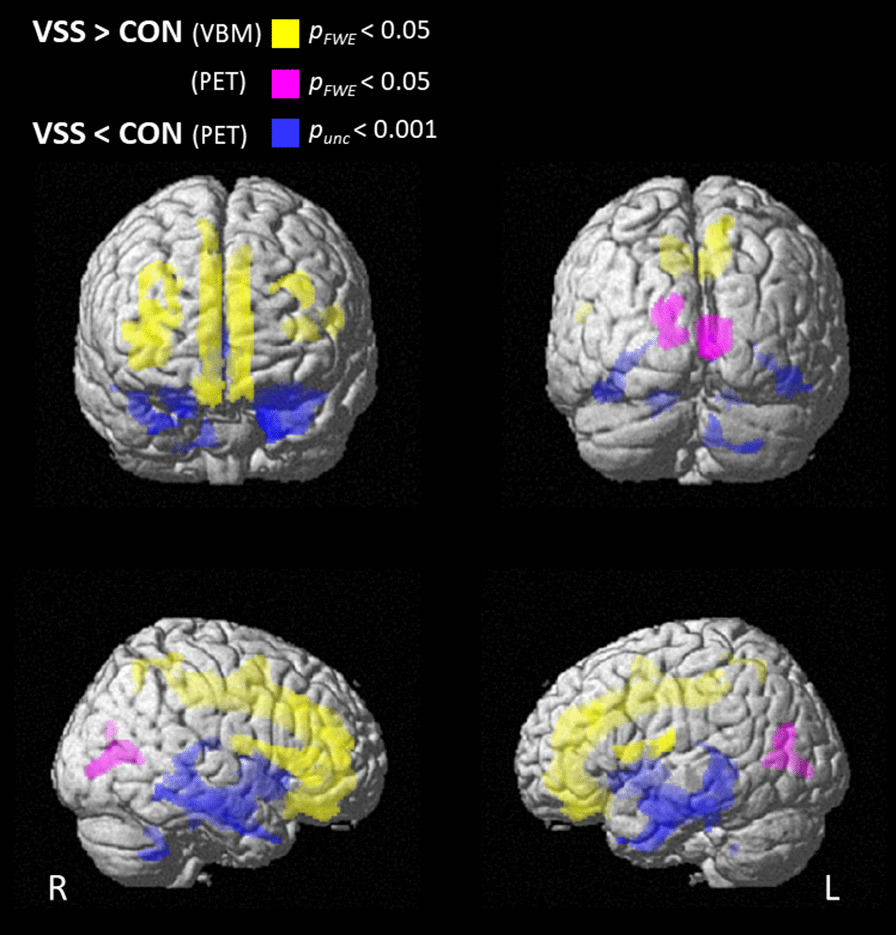
Differences in brain grey matter volume GMV (voxel-based morphometry, VBM) and glucose metabolism (PET) between patients with visual snow syndrome (VSS) and controls (CON). Surface rendering showing brain regions with increased grey matter volume (yellow), increased glucose metabolism (purple) (*p*_FWE_ < 0.05) and decreased glucose metabolism (blue; *p*_uncorrected_ < 0.001) in VSS compared to CON

**Fig. 4:**
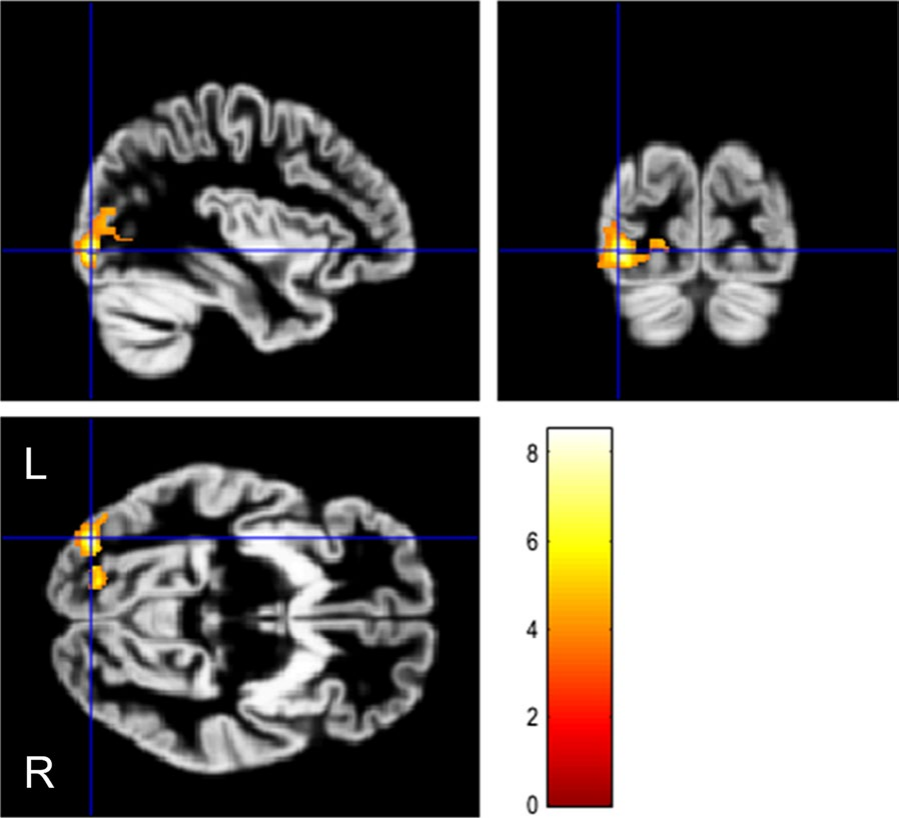
VBM analysis with small volume correction restricted to the occipital lobe, showing the increase of gray matter volume in visual snow syndrome (VSS) patients compared to CON. SPM displayed at *p*_height_ < 0.001 uncorrected and *K*_ext_ > 200 voxels. Clusters are overlaid onto the average GM from the whole study population. Significance is shown with a T statistic color scale. Images are in neurological orientation

VSS patients showed no differences in average total intracranial volume (TIV) with respect to controls (mean ± SEM 1578 ± 41 cm^3^ vs. 1515 ± 60 cm^3^). Whole-brain VBM analysis showed widespread clusters of increased GM volume in VSS patients, symmetrically distributed in the insula, prefrontal cortex (inferior, middle, and superior frontal gyrus), anterior and middle cingulum (*p*_FWE_ < 0.0001 at cluster level, range *T* values: 8.4–17.5; Fig. 3 and Additional file 1: Table S1). Only on a lower *p* threshold (*p*_height_ < 0.001 uncorrected), and by applying a mask corresponding to the main occipital regions with PET abnormalities (see previous paragraph and Table 2A1–B1; generated in WFU PickAtlas SPM toolbox), VBM identified subtle GMV increases in the lingual gyrus and left lateral occipital gyrus (secondary V2 and associative visual cortex V3, V4, V5) of VSS patients (range *T* values: 6.1–8.6; Fig. 4). No region displayed lower GMV in VSS compared to CON.

The VOI MR GMV group comparison confirmed GMV increases in VSS patients in the frontal cortex (*p* = 0.001) and cingulate cortex (*p* < 0.001) (all Bonferroni corrected).

### Discriminant analysis using ^18^F-FDG PET and GMV

Discriminant analysis was performed for regional ^18^F-FDG and GMV VOI values separately, as well as by combining the 2 data sets. Using ^18^F-FDG VOIs, 100% of cross-validated grouped (VSS and CON) cases were correctly classified by the discriminant model (Table 3A). Among the VOIs most significantly retained in the discriminant model (Wilks´ Lambda discriminant function = 0.014; *p* < 0.001) were the lingual gyrus (AUC = 0.95), the cuneus (AUC = 0.92), and the lateral occipital cortex (AUC = 0.84).

**Table 3:** ^18^F-FDG PET and structural MR-based grey matter volume (GMV) classification accuracy based on the discriminative VOI-based analysis (A) and visual read analysis of two blinded experienced observers (B)

	Group	Predicted Group Membership		Total
		CON	VSS	
*(A) Discriminant analysis of VOI-based *^*18*^*F-FDG PET and MR GMV, after leave-one-out cross-validation*				
FDG				
Count	CON	15	0	15
	VSS	0	7	7
%	CON	100	0	100
	VSS	0	100	100
GMV				
Count	CON	14	1	15
	VSS	1	6	7
%	CON	93	7	100
	VSS	14	86	100
FDG + GMV				
Count	CON	15	0	15
	VSS	1	6	7
%	CON	100	0	100
	VSS	14	86	100

Final diagnosis	PET “VSS pattern”		PET normal	
	Observer 1	Observer 2	Observer 1	Observer 2
*(B) Visual classification using *^*18*^*F-FDG PET*				
CON	0	6	15	9
VSS	5	1	2	6

CON = healthy controls; VSS = patients with visual snow syndrome

Performing the discriminant analysis by using MR GMV values resulted in a 91% accuracy for differentiating VSS and CON (Wilks’ Lambda discriminant function = 0.06, *p* < 0.001). In contrast to ^18^F-FDG PET data, the most significant discriminative VOIs for GM volumes were located in more widespread cortical areas, with the cingulum (AUC = 1.00), the frontal lobe (AUC = 0.91), and the parietal lobe (AUC = 0.85) increases as most significant.

Combined information with an independent entering for both ^18^F-FDG and GMV resulted in an accuracy of 95% for the differentiation of CON and VSS patients (Table 3A).

### Visual ^18^F-FDG PET analysis

The classification accuracy of the visual read of ^18^F-FDG PET for the two observers is given in Table 3B. A substantial difference between observers was noted in both sensitivity and specificity, both in assessing hypermetabolism in the lingual gyrus as well as the hypometabolism in the mesotemporal cortex for the VSS group (Additional file 1: Fig. S2). The first observer correctly identified 5 out of 7 patients as pathological (VSS pattern) (sensitivity = 71%) and the 15 CON as normal (specificity = 100%; accuracy = 91%), with an average confidence rating of 3.1 (*reasonably certain*) for VSS and 3.8 (*certain*) for CON. The second observer identified 1 out of 7 patients as pathological (sensitivity = 14%) and 9 controls as normal (specificity = 60%; accuracy = 45%), with a degree of diagnostic confidence equals to 3.4 and 3.1 respectively (*reasonably certain*) for both VSS and CON.

## Discussion

Patients with visual snow syndrome, defined according to the consensus criteria [4], show highly significant metabolic and structural differences when compared to a screened group of age-matched healthy volunteers. As main finding, we observed relative hypermetabolism in the lingual gyrus, but this hypermetabolism was also extending to the cuneus and secondary associative visual cortex. Also, the right cerebellar lobules Crus 2–VIIB/VIII were metabolic more active at rest in VSS patients. These metabolic findings are consistent with the only previously published FDG series in 20 VSS patients, where a more spatially restricted hypermetabolism was found in the right lingual gyrus and upper edge of the (left) cerebellar anterior lobe adjacent to the left lingual gyrus [13, 21]. Apart from metabolic increases in the visual cortex, we also observed hypometabolism to the medial temporal cortex. The findings were corroborated by VOI based analysis. The intensity differences were robust enough so that discriminant analysis of the relative ^18^F-FDG uptake data was able to fully separate VSS patients without overlap (see Fig. 1C). The voxel-based cluster intensity differences in the lingual gyrus and cuneus were of the order 10–20% (peak value 24%), therefore we also investigated if this enabled visual assessment at the individual level. However, this proved to be difficult and variable between even experienced observers.

Overall, the metabolic findings in this study confirm the particular role for the lingual gyrus in VSS. Hypermetabolism in VSS seems specific compared to migraine, which is one of the main comorbidities in VSS. Inclusion of the presence of migraine as covariate did not alter the group results. Also, in migraine alone, studies were not able to show hypermetabolism in interictal migraineurs in comparison to non-migraineur controls [21]. Even though our sample size was small, the high spatial resolution of the Signa PET/MR data (about 4 mm) and time-of-flight acquisition, increases signal to noise and careful acquisition monitoring (especially the visual context with dark environment during ^18^F-FDG administration in VSS and CON groups) may have led to a high sensitivity for these abnormal findings in these cortical areas despite the relatively small VSS group.

The underlying mechanism for lingual hypermetabolism at rest can be a combination of hyperexcitation due to neural hypersensitivity [17] and underlying grey matter increases [13, 22]. As grey matter increases in the lingual gyrus were only modest in this patient group (only after small volume correction in VBM), our finding indicates that functional hyperexcitability may be a more dominant driver of the observed increase in metabolic activity.

The hypermetabolic cluster observed in the right cerebellum may be in concordance with a role attributed to the cerebellum in visuomotor activity, including visual guidance of movement, control of smooth pursuit (attention regulation) and sensorimotor or visuomotor adaptation [29, 30], that may be altered due to the bias induced in the added visual perception by VSS.

The finding of reduced metabolism in the hippocampus and parahippocampal region, is at odds with Schankin et al. [13], who found modest hypometabolism in the inferior parietal and lateral superior temporal cortex. Although a difference in patient type or comorbidity may be present, past recreational drug use and presence of anxiety/depression are not typically related to hypometabolism of the mesial temporal cortex. Although a structured symptom questionnaire was included in the workup of all patients, no elaborated neuropsychological patient profiling was conducted and memory domains were not specifically questioned or tested as less prevalent in VSS. A connection with memory complaints could therefore not be made. In contrast, prefrontal, anterior cingulate hypometabolism and mesial temporal hypermetabolism is mainly associated with anxiety and major depression [31, 32]. Also, VS intensity differences may have played a role, as it is known that VSS represents a continuum in intensity, with higher intensity more likely to be associated with comorbidities such as tinnitus and migraine [12].

As for grey matter volume differences, we found a more widespread increase in GMV for VSS patients, in the prefrontal, cingulate and parietal areas. Only when applying a small volume correction, also in the lingual gyrus and secondary visual cortex, an increase in regional GMV was observed. These results for GMV are showing more extensive involvement in VSS than found by Schankin et al. [13], Pulleda et al. [22] and Aldusary et al. [23], where apart from the visual cortex some increases in the temporal and limbic cortex were seen. Also here, extravisual symptomatology may have contributed to these differences. For example, GM volume increase in the prefrontal cortex has been observed in patients with chronic migraine [33, 34], although this is not a consistent finding [35, 36]. The prefrontal cortex has extensive connections with limbic and sub-cortical areas and is involved in pain perception and cognitive-emotional processing. Involvement of the insula in VSS has been demonstrated recently using BOLD fMRI [14]. The anterior insula is essential for selecting information that is relevant for the brain and conveys the information deemed significant to other areas of the limbic system. In VSS, stimuli such as the intrinsic “snow” that should normally be considered irrelevant “pass” a certain salience threshold, finally turning into an apparently normal perception.

Although VSS diagnosis is based on clinical criteria, this and previous work has indicated that marked imaging findings are associated in VSS patients. Clinical diagnostic accuracy of the Schankin criteria is still unknown, and further validation and criteria refinement is ongoing. However, in view of the robustness of the imaging findings in the lingual gyrus, their specificity (as not typically found in migraine or migraine aura, the most prevalent comorbidities [13]), and high sensitivity in detecting VSS subjects on an individual level, imaging may play an important adjunct role on an individual patient level. Semiquantitative FDG uptake in the lingual gyrus, cuneus and lateral occipital cortex was found to provide excellent classification accuracy, but the value of the discriminant function should be investigated in an independent dataset. Visual assessment of these differences by readers resulted in reasonable average specificity of 0.80 but low mean sensitivity 0.43. For most clinical syndromes where ^18^F-FDG PET is used (dementia, movement disorders, epilepsy, traumatic brain injury), detailed analysis of abnormalities within the visual cortex is not typically performed and is also depending on injection circumstances (ambient light). This may in part contribute to the relative insensitivity of the visual analysis and interrater dependence. For potential future clinical use, VOI semiquantification with discriminant analysis and comparison to a normal dataset is therefore preferred.

The major limitations of this study include the small sample size of the patient population. This also means that, for now, findings outside the lingual gyrus as reported by us should be interpreted with a bit more caution and need further confirmation, and in case of doubt the larger study by Schankin et al. remains the primary reference [13]. Second, 71% of the patients presented with migraine or tinnitus, the two most common comorbid conditions in visual snow syndrome. Although there is evidence that these conditions are independently associated with a more severe presentation of the syndrome, our findings point to similar metabolic changes [22]. Six out of seven patients presented with psychiatric symptoms including anxiety, depression, depersonalization and compulsive neurosis. Third, two patients (# 2 and 5, Table 1) did not suffer a *continuous* panfield visual disturbance, which was defined as original criterion for VSS by Schankin [4]. Visual snow syndrome was here an exclusion diagnosis as neurological, ophthalmological and paraclinical findings could not identify another underlying cause for their symptoms.

Last, a history of cannabis or recreational drug use was reported by five patients. Hallucinogen‑Persisting Perception Disorder (HPPD), mostly occurring after XTC or cannabis use, can present with features similar to VSS with spontaneous recurrence of visual perceptual disorders separated in time from the initial exposure [37]. Although this condition may somewhat resemble to visual snow, HPPD-associated hallucinations rather consist of geometric shapes, peripheral vision disturbances and flashes of different colors [37], unlike the typical snow-phenomenon that was experienced by our patient group. Furthermore, four out of these five patients stopped using any drugs at least 8 months prior to PET/MR imaging. One patient with ongoing active cannabis/recreational drug reported that his VS symptoms were not related to usage. Moreover, similar results were observed when the latter patient (#7, Table 1) was excluded from the group analysis. Therefore, it is unlikely that overall the reported abnormalities are mere secondary to the use of recreational substances.

While our study and several recent ^18^F-FDG PET and advanced MRI reports are starting to clarify the biological basis of VSS, both in terms of regional neuronal activity and network dysfunctions such as the salience network, the observed findings can be an epiphenomenon or direct contributors to the mechanism of VS. In order to dissect the etiology of visual snow alone versus the secondary phenomena present in VSS such as palinopsia, entoptic symptoms, nyctalopia or photophobia, as well as impact of comorbidities such as tinnitus and migraine/migraine aura, more study power is needed with preferably a multimodal, multiparametric approach and with clinical profiling of visual, auditory, emotional, and cognitive functions. This may then offer directions for potential therapeutic developments for this currently very challenging to manage disorder.

In conclusion, patients with VSS show consistent metabolic changes in the associative visual cortex, as well as significant metabolic and structural abnormalities that are not only confined to the visual system. All VSS patients were correctly classified by a discriminant analysis using relative FDG uptake values, which slightly outperforms MR volumetry. Visual analysis of FDG PET is, however, much less performant. Further research is needed to distinguish the different but related disorders of VSS, migraine and tinnitus.

## Supplementary Information

**Additional file 1. Table S1**. Cluster peak locations of the unpaired t-test performed for the voxel-based morphometry (VBM) analysis. L = left; R = right. **Fig. S1** SPM 12 surface rendering showing the decreased glucose metabolism in visual snow syndrome (VSS) patients compared to healthy controls (CON) when we covaried for migraine only (dark blue) and for migraine and tinnitus (light blue) (p_height_ < 0.005 uncorrected). **Fig. S2** Visual intensity rating for 18F-FDG PET for both observers for left (L) and right (R) medial temporal (mesotemporal) cortex and lingual gyrus for the diagnostic group of visual snow syndrome (VSS) patients. Data bars indicate with mean and range. Y-axis shows the visual rating: -2 = strongly decreased, -1 = slightly decreased, 0 = normal, 1 = slightly increased, 2 = strongly increased). Open circles indicate those VSS patients who were misclassified, closed the correct classification.

## Data Availability

Anonymized data will be deposited in an access-controlled file server used by the academic research PET imaging group, which will be shared on reasonable request from any qualified investigator on approval by the Ethics Committee of the local university hospital.
