# Supplementary material for: Simultaneous 18F-FDG PET/MR metabolic and structural changes in visual snow syndrome and diagnostic use
Source: EJNMMI Res. 2022 Dec 30;12:77. doi: 10.1186/s13550-022-00949-0 (PMC9803799; doi:10.1186/s13550-022-00949-0)

# Additional file

**Additional file 1: Table S1**

Cluster peak locations of the unpaired t-test performed for the voxel-based morphometry (VBM) analysis. L = left; R = right.

| Cluster level | | | Voxel level | | | | Peak voxel  Talairach coordinate | | | | Cluster  location | |
| --- | --- | --- | --- | --- | --- | --- | --- | --- | --- | --- | --- | --- |
| P_FWE-corr_ | K_ext_ | | P_FWE-corr_ | T score | | | X | | Y | Z | Anatomical  region | |
| VSS > CON (*p*_height_ < 0.05, FWE-corr) | | | | | | | | | | | | |
| < 0.0001 | 1871 | < 0.0001 | | | 17.5 | 32 | | 26 | | 8 | | R Insula |
|  |  | < 0.0001 | | | 11.1 | -12 | | -68 | | 8 | | R inf front gyr |
| < 0.0001 | 8833 | < 0.0001 | | | 17.4 | 3 | | 8 | | 35 | | R mid cing cx |
|  |  | < 0.0001 | | | 17.0 | 8 | | 0 | | 38 | | R mid cing cx |
|  |  | < 0.0001 | | | 17.0 | -2 | | 24 | | 27 | | L ant cing cx |
| < 0.0001 | 360 | < 0.0001 | | | 11.0 | -50 | | -11 | | 20 | | L postcentr gyr |
| < 0.0001 | 241 | 0.001 | | | 8.7 | -36 | | 29 | | 27 | | L inf front gyr |
|  |  | 0.012 | | | 7.3 | -29 | | 30 | | 33 | | L mid front gyr |
|  |  | 0.034 | | | 6.6 | -23 | | 39 | | 30 | | L sup front gyr |
| < 0.0001 | 340 | 0.002 | | | 8.4 | -39 | | 9 | | 14 | | L front inf oper |

**Additional file 1: Fig. S1**

SPM 12 surface rendering showing the decreased glucose metabolism in visual snow syndrome (VSS) patients compared to healthy controls (CON) when we covaried for migraine only (dark blue) and for migraine and tinnitus (light blue) (p_height_ < 0.005 uncorrected).


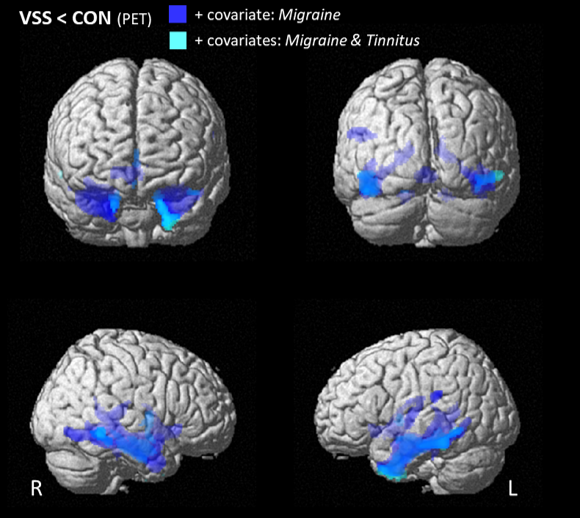


**Additional file 1: Fig. S2**

Visual intensity rating for ^18^F-FDG PET for both observers for left (L) and right (R) medial temporal (mesotemporal) cortex and lingual gyrus for the diagnostic group of visual snow syndrome (VSS) patients. Data bars indicate with mean and range. Y-axis shows the visual rating: -2 = strongly decreased, -1 = slightly decreased, 0 = normal, 1 = slightly increased, 2 = strongly increased). Open circles indicate those VSS patients who were misclassified, closed the correct classification.


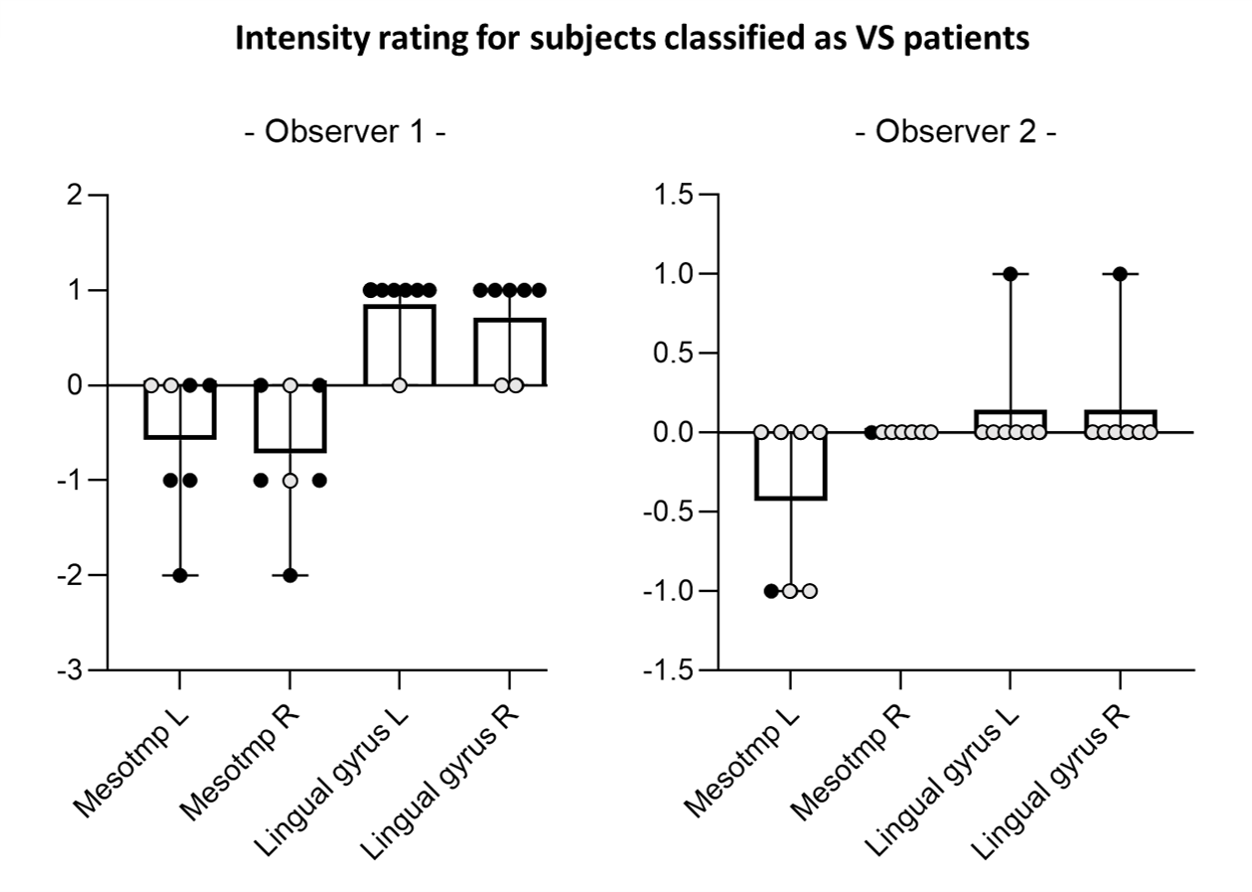

Supplement: Supplementary file 1 — Additional file 1. Table S1. Cluster peak locations of the unpaired t-test performed for the voxel-based morphometry (VBM) analysis. L = left; R = right. Fig. S1 SPM 12 surface rendering showing the decreased glucose metabolism in visual snow syndrome (VSS) patients compared to healthy controls (CON) when we covaried for migraine only (dark blue) and for migraine and tinnitus (light blue) (pheight < 0.005 uncorrected). Fig. S2 Visual intensity rating for 18F-FDG PET for both observers for left (L) and right (R) medial temporal (mesotemporal) cortex and lingual gyrus for the diagnostic group of visual snow syndrome (VSS) patients. Data bars indicate with mean and range. Y-axis shows the visual rating: -2 = strongly decreased, -1 = slightly decreased, 0 = normal, 1 = slightly increased, 2 = strongly increased). Open circles indicate those VSS patients who were misclassified, closed the correct classification. [file 13550_2022_949_MOESM1_ESM.docx]
